# Immune checkpoint-induced arthritis: a comprehensive single-cohort descriptive analysis from clinical evaluation to histology

**DOI:** 10.3389/fmed.2025.1638139

**Published:** 2025-10-13

**Authors:** Laurent Meric de Bellefon, Francesco Natalucci, Adrien Nzeusseu Toukap, Tatiana Sokolova, Christine Galant, Frank Aboubakar, Jean-François Baurain, Frank Cornélis, Ivan Borbath, Patrick Durez

**Affiliations:** ^1^Rheumatology Department, Cliniques Universitaires Saint-Luc, Brussels, Belgium; ^2^Pôle of Systemic and Inflammatory Rheumatic Pathologies, Institut de Recherche Expérimentale et Clinique, Université Catholique de Louvain, Brussels, Belgium; ^3^Department of Pathology, Cliniques Universitaires Saint-Luc, Brussels, Belgium; ^4^Division of Pneumology, Cliniques Universitaires Saint-Luc, Brussels, Belgium; ^5^Department of Medical Oncology, Université Catholique de Louvain and Cliniques Universitaires Saint-Luc, Brussels, Belgium; ^6^Department of Gastroenterology, Cliniques Universitaires Saint-Luc, Brussels, Belgium

**Keywords:** immune checkpoint inhibitors-induced arthritis, ICIs-IA, rheumatic immune-related adverse event, irAEs, ultrasound, US, guided synovial biopsy, USGSB

## Abstract

**Background/purpose:**

Immune checkpoint inhibitor-induced arthritis (ICI-IA) is the most common rheumatic immune-related adverse event (irAEs). Its pathogenesis remains unknown. Ultrasound (US)-guided synovial biopsy (USGSB) has been proven to be a safe and reliable procedure in rheumatoid arthritis (RA), enlarging the understanding of synovitis in RA. To the best of our knowledge, no studies have analyzed the histology of ICI-induced arthritis. This study aimed to describe ICI-IA from clinical presentation to histology.

**Materials and methods:**

Patients who developed inflammatory arthritis while under treatment by ICIs were enrolled. A US assessment of 38 joints [shoulders, elbows, knees, wrists, metacarpophalangeal joints (MCPs), proximal interphalangeal joints (PIPs), and metatarsophalangeal joints (MTPs)] was systematically performed for inflammatory assessment and joint selection before synovial biopsy. Histopathological analyses consisted of semiquantitative scores for histological parameters (synovial hyperplasia, fibrinoid necrosis, hypervascularization, and inflammatory infiltrate) and immunohistochemistry staining (CD3, CD20, CD138, and CD68). As a control group, we enrolled age- and sex-matched untreated early RA (ERA) patients with synovial tissue available.

**Results:**

A total of 13 patients were included [men/women (10/3), with a median age of 65 years (interquartile range: IQR: 14.5)]. Overall, seven patients suffered from polyarthritis (53.8%), and five (38.4%) suffered from oligoarthritis. The US pre-biopsy evaluation detected synovitis in 23.4% of joints; the most involved was the knee, followed by wrist, elbow, MCP2, MCP3, and MTP 2–5. Samples for synovial tissue analysis were primarily obtained from the knee (69.2%). The histology of ICI-induced arthritis demonstrated a synovial inflammation similar to that found in ERA. ICI-IA and ERA show similar clinical and histological characteristics.

**Conclusion:**

Clinically, ICI-IA manifested as oligoarthritis and polyarthritis, similar to RA. Synovial histology in ICI-induced arthritis is indistinguishable from RA, suggesting common pathogenic mechanisms. Further transcriptomics analyses are ongoing to better describe this new arthritis condition.

## Introduction

Immune checkpoint inhibitors (ICIs) have revolutionized the treatment and prognosis of cancer (1). ICIs are monoclonal antibodies against key regulatory proteins such as programmed death 1 and its ligand (PD1-PDL1) or cytotoxic T-lymphocyte-associated protein 4 (CTLA4), enhancing the immune response against cancer cells. Preventing CTLA-4 from binding to its ligands can disrupt immune checkpoint signaling and reduce Treg-mediated immunosuppression. Anti-PD-1 or anti-PD-L1 antibodies reverse the inhibitory signals sent to T cells and activate them. The ICIs acts on their targets, which are the immune cells and, more precisely, on T cells, but the desired effect is achieved indirectly on tumor cells. Reversing the cancer inhibition by ICIs increases the survival rate but exposes the patients to side effects. ICI toxicity refers to a range of inflammatory manifestations collectively termed immune-related adverse events (irAEs). They vary from mild and self-limiting to severe and potentially life-threatening manifestations (2). Rheumatic irAEs are among the most common irAEs and include the development of ICI-induced arthritis (ICI-IA) (3) in 1–7% of the treated patients (4). From a clinical perspective, it usually appears in the first 6 months after ICI initiation, without a gender ratio, as a seronegative oligo-polyarticular arthritis in the absence of rheumatoid factor (RF) or anti-citrullinated protein antibodies (ACPA) (5). Its pathogenesis is largely unknown: the obvious connection with ICIs suggests an immune pro-inflammatory reaction potentially related to T-cell activation (6).

Synovial tissue analysis has been developed in recent years and can be easily obtained by knee mini-arthroscopy and, more recently, by ultrasound (US)-guided synovial biopsies (USGSB). In recent times, the latter procedure is a widespread technique allowing the collection and analysis of high-quality samples of synovial tissue (ST) in various infectious, inflammatory, and non-inflammatory conditions (7). Our group and others have developed studies to analyze the synovial tissue in this early phase of RA (ERA) (8, 9). Different types of synovial pathotypes are described based on the cell types of the immune infiltration. Despite the potential differences between ERA and ICI-IA, some clues link the two. Recent studies have shown that synovial infiltrating T cells in RA patients are PD1-positive and correlate with disease activity. Still, at the same time, PD-L1 is poorly expressed in synovial tissue (10, 11). Thus, an agonistic action on PD1 in RA patients is believed to inhibit T cells. A first phase two trial with peresolimab has recently suggested a potential effect of anti-PD1 in RA (12).

This study aimed to provide a histopathological assessment of ICI-IA tissue, with a further comparison with a control cohort of untreated ERA patients.

## Patients and methods

### Patients

Oncologic patients who were treated with ICIs and had signs and/or symptoms of arthritis were referred to our rheumatologic department. Each patient underwent a complete rheumatological evaluation, including tender joint count (TJC), swollen joint count (SJC), disease activity score 28 joints (DAS28), Health Assessment Questionnaire (HAQ), and research of autoantibodies [anti-nuclear antibodies (ANA), RF, and ACPA].

### Ultrasound assessment and ultrasound-guided synovial biopsies

Musculoskeletal ultrasound (US) assessment was performed using a Logiq E9 machine (GE Healthcare, Chalfont St. Giles, Buckinghamshire, United Kingdom) equipped with a 6–15 multifrequency linear array transducer. The US evaluation included 38 joints [shoulders, elbows, knees, wrists, metacarpophalangeal joints (MCPs), proximal interphalangeal joints (PIPs), and metatarsophalangeal joints (MTPs)]. Patients underwent synovial biopsies either by USGSB or mini-arthroscopy (mini-A). The biopsy location was decided after a systematic US evaluation, according to the presence of synovial hypertrophy, safety, and feasibility. Synovitis was defined according to the OMERACT guidelines (13) as follows:

- Grade 0: no grayscale-detected SH and no PD signal.
- Grade 1 (minimal synovitis): Grade 1 SH and ≤Grade 1 PD signal.
- Grade 2 (moderate synovitis): Grade 2 SH and ≤Grade 2 PD signal.
- Grade 3 (severe synovitis): Grade 3 SH and ≤Grade 3 PD signal.

Synovitis was then defined as the presence of at least a Grade 1 SH. Moreover, to ensure a minimal quality for synovial tissue analysis, at least a Grade 2 SH was required to perform a biopsy.

At least four synovial tissue samples were collected from small joints and six from large joints to ensure adequate material for both histological evaluation and research purposes. The procedure and the tissue analysis were performed according to the European Alliance of Associations for Rheumatology (EULAR) minimal report requirements for synovial tissue research (14). As controls, we selected patients from a cohort of ERA with synovial tissue available, matched for age and sex. The study was approved by the local ethical committee (PISCO No. P1200-502019/27NOV/526), and signed informed consent was obtained from all patients.

### Histological and immunohistochemical evaluation

Tissue samples were fixed overnight in 10% formalin buffer at pH 7.0 and embedded in paraffin for histology and immunohistochemistry. Four parameters were assessed on hematoxylin eosin (H&E) slide: (1) degree of lining layer hyperplasia, scored according to the increase in the number of lining cell layers (with/without multinucleated cells); (2) intensity of inflammatory infiltrate, according to the presence, abundance, and distribution of inflammatory infiltrate (including the presence of lymphocytes and follicle-like aggregates); (3) fibrinoid necrosis (FN), defined as the presence of fibrin deposition within and around the capillaries and/or small vessels; (4) hypervascularization, evaluated as an increased number and/or size of vascular cross-sections per analyzed section. Each parameter was scored on a 7-level semiquantitative scale (0–3, in 0.5 increments) by an expert pathologist blinded to clinical data (CG) (15, 16).

Similarly, with the same scoring system [0–3], the degree of immune cell infiltration was determined through immunohistochemical staining for B cells (CD20) [CD20 Biocare Medical Clone L26, dilution 1:200], T cells (CD3) [CD3 Roche Clone 2GV6], macrophages (CD68) [CD68, Dako clone PGM1 1:40], and plasma cells (CD138) [CD138 Dako clone M& 15 1:20].

### Statistical analysis

All analyses were performed using SPSS 26.0.0 and GraphPad 9.0. Normally distributed variables were summarized using the mean (SD), and non-normally distributed variables were summarized using the median and range (IQR). Frequencies were expressed as percentages. Wilcoxon’s matched pair test and paired t-test were performed accordingly. Univariate comparisons between nominal variables were calculated using the chi-squared test or Fisher’s exact test where appropriate. Spearman’s test was used to assess the correlations. Two-tailed *p*-values were reported, and *p*-values of less than 0.05 were considered significant.

## Results

### Clinical evaluation

We included 13 patients [men/women (10/3), with a median age of 65 years (IQR 14.5)]. Clinical, serological, and demographic characteristics are summarized in Table 1, while Table 2 provides an extensive report on each patient profile. Only one patient had a previous diagnosis of rheumatic disease (RA), while two suffered from psoriasis. Malignancies were hepatocellular carcinoma (*n* = 2), kidney carcinoma (*n* = 2), small-cell lung carcinoma [SCLC] (*n* = 1), non-SCLC (*n* = 2), non-melanoma skin cancer (*n* = 1), melanoma (*n* = 4), and endometrial carcinoma (*n* = 1). A total of nine patients received an anti-PD1, and four patients received an anti-PD-L1, while two patients received a combination therapy with CTLA4 inhibitors.

**Table 1:** Clinical, serological, and demographic features of ICI-IA and ERA.

	ICI-IA	ERA	*p*
	*N* = 13	*N* = 22	
Men/women	10/3 (76.7/23.9)	17/5 (77.2/22.7)	–
Previous AID	3 (23)	–	–
Median (IQR)			
Age (years)	65 (14.5)	62.59 (14.1)	0.89
Time of onset (weeks)	5 (17)	3 (9)	0.30
TJC	3.5 (8)	5 (7.5)	0.51
SJC	5 (6)	6 (8.5)	0.11
HAQ	1.06 (1.09)	1.62 (0.84)	0.32
CRP (mg/dL)	2.95 (10.1)	2.8 (4.7)	0.65
DAS28	5.48 (1.43)	5.72 (2.11)	0.79
Autoantibodies N (%)			
ANA	4 (36.4)	–	–
Rheumatoid factor	2 (18.2)	10 (45.4)	0.13
ACPA	0 (0.0)	12 (54.4)	0.0009
Cancer histology N (%)			
Melanoma	4 (30.7)	–	–
Kidney	2 (15.3)	–	–
Skin	1 (7.7)	–	–
NSCLC	2 (15.3)	–	–
SCLC	1 (7.7)	–	–
HCC	2 (15.3)	–	–
Endometrial carcinoma	1 (7.7)	–	–
Immune checkpoint inhibitors N (%)			
Monotherapy 9 (69.2)			
Pembrolizumab	6 (46.1)	–	–
Cemiplimab	1 (7.7)	–	–
Durvalumab	2 (15.4)	–	–
Combo-therapy N (%)			
Ipilimumab–nivolumab	2 (15.4)	–	–
Atezolizumab–bevacizumab	2 (15.4)	–	–
Histology			
Sublining hyperplasia	0.90 ± 0.88	0.85 ± 0.67	0.86
Inflammatory infiltrate	1.30 ± 0.67	1.13 ± 0.83	0.56
Fibrinoid necrosis	1.00 ± 0.81	1.35 ± 1.18	0.40
Hypervascularization	0.60 ± 0.70	1.48 ± 1.83	0.15
CD3	1.20 ± 0.63	1.09 ± 0.85	0.72
CD20	0.55 ± 0.69	0.45 ± 0.63	0.70
CD138	0.60 ± 0.61	0.53 ± 0.65	0.78
CD68	1.15 ± 0.58	1.48 ± 0.72	0.21
CD68 necrosis score	2.15 ± 0.94	2.80 ± 1.77	0.29

AID, autoimmune diseases; TJC, tender joint count; SJC, swollen joint count; DAS28, Disease Activity Score 28 joints; CRP, C-reactive protein; ACPA, anti-citrullinated protein antibodies; ANA, anti-nuclear antibodies; ANCA, anti-neutrophil cytoplasm antibodies; NSCLS, non-small-cell lung carcinoma; SCLS, small-cell lung carcinoma; HCC, hepatic carcinoma; CD3, lymphocytes; CD20, B cells; CD68, macrophages; CD138, plasma cells. Histological assessment expressed as mean value ± standard deviation. CD68 necrosis score, composite score derived from the sum of CD68 and fibrinoid necrosis amount assessment. Considering a (0–3) grading scale for both variables, the CD68 necrosis score may vary in the (0–6) range.

**Table 2:** Patients’ description.

Patients	Sex	Age	Malignancy	ICIs	Previous AIDs	Time-to-onset (weeks)	TJC	SJC	Ultrasound synovitis	Involved joints (size)	Articular pattern
1	M	59	Hepatocarcinoma	Atezolizumab–bevacizumab	–	3	5	1	18	Small/medium/large	Polyarthritis
2	M	72	Melanoma	Pembrolizumab	–	1	12	10	11	Small/medium/large	Polyarthritis
3	F	73	Kidney	Pembrolizumab	–	48	16	8	8	Small/medium/large	Polyarthritis
4	M	69	Kidney	Pembrolizumab	Psoriasis	4	2	2	1	Large	Oligoarthritis
5	F	87	Endometrium	Pembrolizumab	–	4	2	6	6	Small	Polyarthritis
6	M	46	Melanoma	Ipilimumab–nivolumab	Psoriasis	6	2	2	2	Large	Oligoarthritis
7	M	60	Non-melanoma skin	Cemiplimab	–	20	2	2	2	Large	Oligoarthritis
8	M	72	Hepatocarcinoma	Atezolizumab-bevacizumab	–	2	5	5	8	Small/medium/large	Polyarthritis
9	F	71	NSCLC	Durvalumab	RA	n.a	4	4	3	Medium/large	Oligoarthritis
10	M	65	Melanoma	Ipilimumab–nivolumab	–	3	2	nd	2	Large	Oligoarthritis
11	M	51	NSCLC	Pembrolizumab	–	20	11	11	4	Medium/large	Oligoarthritis
12	M	65	Melanoma	Pembrolizumab	–	52	7	7	6	Small/medium/large	Polyarthritis
13	M	56	SCLS	Durvalumab	–	12	14	14	28	Small/medium/large	Polyarthritis

ICI, immune checkpoint inhibitors; AIDs, autoimmune diseases; TJC, tender joint count; SJC, swollen joint count; NSCLC, non-small-cell lung carcinoma; SCLC, small-cell lung carcinoma; RA, rheumatoid arthritis.

Overall, seven patients presented a polyarthritis with the involvement of at least four joints. More specifically, four of them showed a symmetric distal arthritis of the hands without involvement of other joints, while the other three also showed medium and/or large joint involvement. In total, five patients presented with an oligoarthritis of medium and large joints and, specifically, two with a symmetric involvement of wrists and knees, two with bilateral knee arthritis, and only one with an asymmetrical involvement of the elbow, wrist, and knee. The last patient developed knee monoarthritis.

Time between the first ICI dose and arthritis development was heterogeneous, as shown in Table 2, with a median of 5 weeks (IQR: 17). A total of two patients receiving a combination therapy developed ICI-IA in the first 6 weeks of treatment, while patients in monotherapy with pembrolizumab also showed late onset of arthritis, up to 1 year after the first ICI administration.

When comparing ICI-IA and ERA, they were similar in terms of disease activity (TJC, SJC, and DAS28) and could only be distinguished by the significantly lower seropositivity for ACPA [0 patients (0%) versus 12 patients (54.4%); *p* = 0.0009]. Overall, 10 patients (76.9%) received glucocorticoids (GCs), one (7.7%) received methotrexate (MTX), and one received tocilizumab as an add-on therapy to GCs.

### Musculoskeletal ultrasound

**Figure 1:**
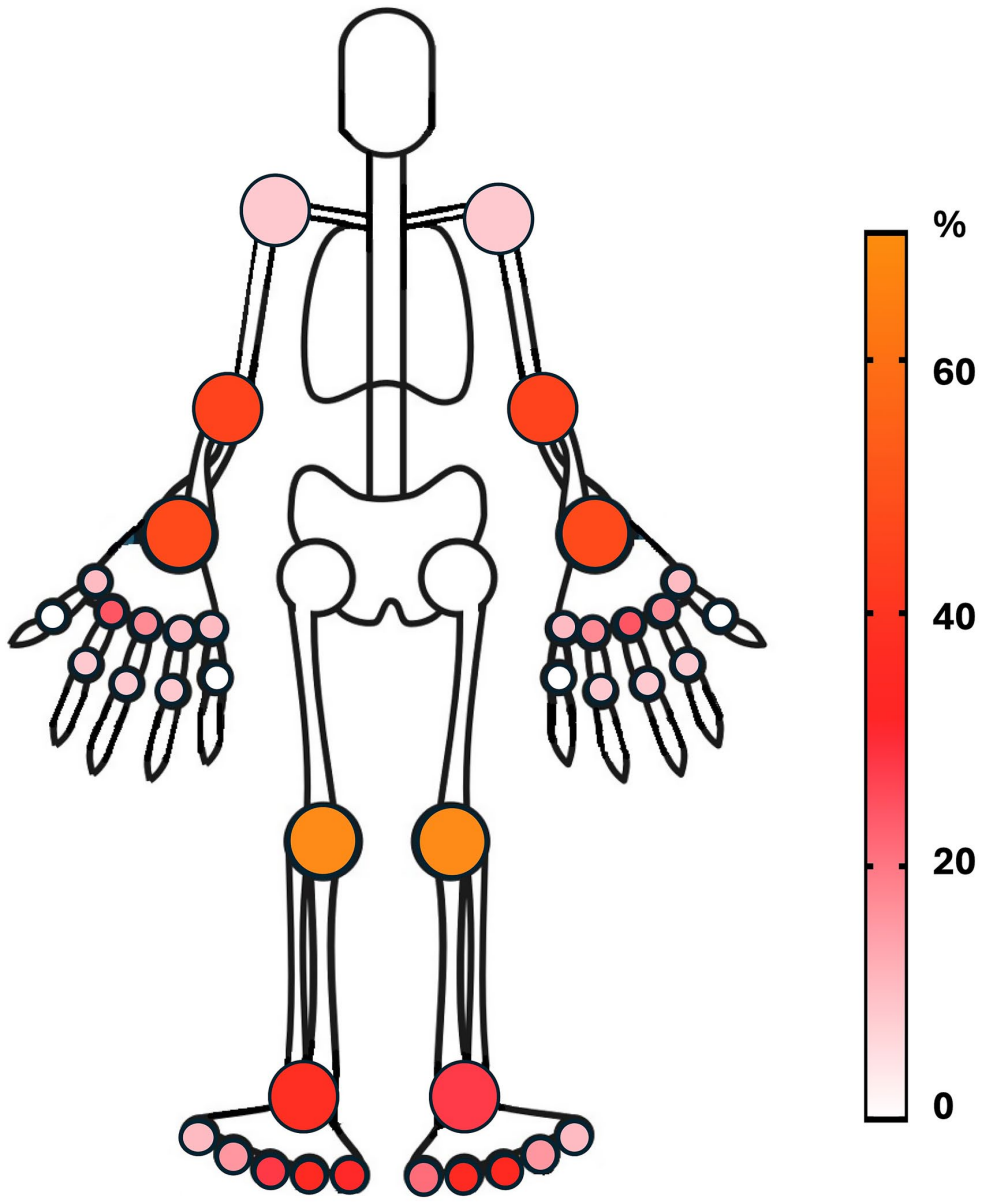
Any Grade (≥1) US-Detected Synovitis. Legend: Each percentage represents the proportion of joints with detected synovitis out of the total number of that specific joint assessed (e.g., left knee: *N* = 9; US-detected synovitis ≥1 = 7; prevalence = 77.7%).

The pre-biopsy ultrasound evaluation (Figure 1) showed the presence of synovitis of any grade (grayscale ≥1) in 23.4% of the joints. The most frequently involved joint was the knee, followed by the wrist, elbow, MCP2, MCP3, and MTP 2–5. The knee was by far the most commonly biopsied joint (69.2%).

Clinical evaluation and US showed generally a good concordance (Cohen’s kappa coefficient of 0.66). However, as for patients No. 1 and No. 3, it was helpful in unraveling the presence of subclinical inflammation: in those cases, US was much more sensitive in detecting inflammation compared to the clinical evaluation.

### Histology and immunohistochemistry

**Figure 2:**
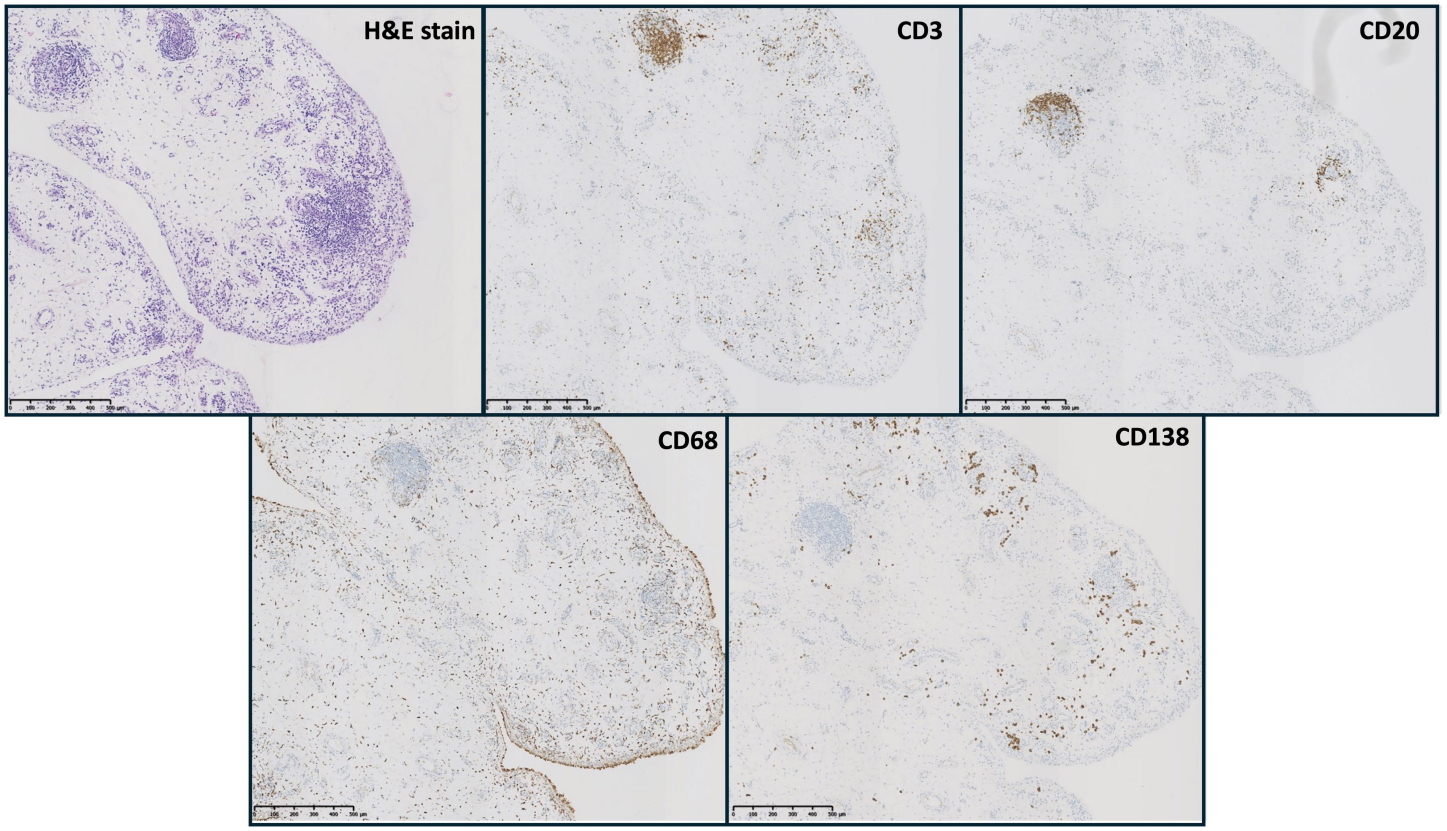
Representative H&E and IHC staining of an ICI-induced arthritis. Legend: H&E, hematoxylin and eosin; CD3, lymphocytes; CD20, B cells; CD68, macrophages; CD138, plasma cells.

The histology of ICI-induced arthritis showed synovial inflammation comparable to that observed in the ERA cohort. This similarity was consistent across all evaluated features, including hematoxylin and eosin (H&E) staining (sublining hyperplasia, inflammatory infiltrate, fibrinoid necrosis, and hypervascularization) and immune infiltrates (CD3, CD20, CD138, and CD68). Representative H&E and IHC images are shown in Figure 2.

We did not identify any significant differences between the variables taken separately and in combination (Table 1).

In a previous synovial study in ERA, we demonstrated how specific histologic features, such as macrophage infiltration and the amount of fibrinoid necrosis, were associated with a more severe phenotype (CD68 necrosis score) and a better treatment response to methotrexate (16). The synovial inflammatory signature does not differ between ERA and ICI-IA, suggesting a similar high synovial inflammatory burden. Histology and IHC results did not correlate with the disease severity indices (DAS28, TJC, and SJC, Supplementary Table 1).

## Discussion

In this study, we have presented the first synovial tissue histological evaluation of an ICI-IA cohort, compared to ERA.

We showed that ICI-IA and ERA share similar clinical and histological characteristics.

From the clinical point of view, ICI-IA has been extensively deciphered (5). It is typically an oligo-polyarthritis involving small, medium, and large joints resembling RA. Our cohort is in line with these observations. Indeed, when comparing ICI-IA with ERA, we did not identify any difference in terms of disease activity (DAS28), joint involvement (TJC and SJC), and serum inflammation (CRP) levels. The only major discriminant was the absence of ACPA antibodies in ICI-IA, confirming the seronegative profile of this condition.

ANAs have been historically associated with cancer as an epiphenomenon, and their prevalence could reach 44.4% also in patients without any signs or symptoms of autoimmune diseases such as systemic lupus erythematosus, Sjogren’s Syndrome, or systemic sclerosis (17–19). More recently, they have been associated with a higher risk of developing irAEs, but more studies are necessary to confirm these observations (20, 21). We observed a prevalence of 36.4% in ICI-IA, which was not different from that found in RA (6/18, 33.3%).

ICI-IA has a favorable outcome and is controlled by corticosteroids and immunosuppressants. bDMARDs have also been positively evaluated in such conditions. Compared to RA, we do not know the long-term course or evolution of ICI-IA. In the synovial tissue analysis, ICI-IA demonstrated significant levels of synovial inflammation, including sublining hyperplasia, inflammatory infiltrate, fibrinoid necrosis, and hypervascularization, all found at similar levels in the ERA cohort. The immunohistochemical analysis further confirmed comparable levels of immune cell infiltration—specifically CD3 + T cells, CD20 + B cells, CD68 + macrophages, and CD138 + plasma cells—between the two groups.

However, it should be underlined that hypervascularization tends to be lower in ICI-IA than in ERA. Hypervascularization is a typical histological feature of different inflammatory arthritides, including RA and Psoriatic Arthritis (PsA) (22). In this study, we show relatively low vascularization in ICI-IA compared to ERA, which is the only feature that differentiates them. While the low sample size requires external confirmation, this finding suggests that a deeper investigation into mechanisms involved in angiogenesis, such as vascular cell adhesion molecule 1 (VCAM-1) and intercellular adhesion molecule 1 (ICAM-1), could unravel differences between the diseases (23).

These similarities regarding clinical appearance and pathology may suggest a common immune activation between the two entities. ICI-IA represents the prototype of inflammatory arthritis and, more generally, of autoimmune diseases: once triggered by an appropriate stimulus, such as ICIs for ir-AEs, the immune system breaks self-tolerance control and targets self-tissues, such as the synovium. On the other hand, RA shares the same pathogenic model, but the triggering stimulus has never been unequivocally demonstrated. However, in addition to the initial trigger, ICI-IA and RA share common ground, including biological pathways and immune dysregulations. PD-1, for instance, plays a central role in both conditions. It is a key regulator of the cellular cycle whose expression on CD4 + and CD8 + lymphocytes reflects T-cell activation, and its binding to PD-L1 and PD-L2 inhibits lymphocytes. Targeting PD1 with blocking antibodies is the rationale behind the development of cancer immunotherapy, leading to the development of several anti-PD1 drugs such as pembrolizumab, nivolumab, and cemiplimab. On the other hand, these same pathways have been shown to play a role in inflammation and autoimmune diseases. Indeed, PD-1 is highly expressed in RA-inflamed synovium, especially on infiltrating T lymphocytes (24, 25), and its targeting showed promising results in a phase two trial (10). The cell cycle regulator CTLA4 shows activity in both directions and has become a therapeutic target in oncology and rheumatology. Ipilimumab and abatacept manipulate the same immune pathway in opposite directions, demonstrating once again extensive overlap of immune mechanisms in cancer and autoimmunity (26).

Despite the growing interest in ICI-IA and ir-AEs in general, knowledge about them is still scarce. However, some studies suggest a prominent role of T lymphocytes over B cells, which may also explain the extremely low ICI-IA seropositivity for ACPA in ICI-IA. More specifically, shared CD8 T-cell clones may be found between the tumor, the lymph nodes, the blood, and the inflamed tissue in cases of irAEs (27, 28). We know now that “toxic” CD8 T cells can also arise directly on-site (29). The specificity of these pathogenic lymphocytes has recently been better characterized as a disease-specific T CD8 subpopulation (CD38high) present both in ST and blood.

On the other hand, RA is a complex disease with numerous determinants, including a key role for B cells and autoantibodies. However, evidence also supports a T-cell-driven mechanism in its pathogenesis: shared T-cell clones have been identified between the joints and lungs of RA patients with interstitial lung disease (30), as well as among inflamed joints within individual RA patients (31). This finding suggests that ICIs may activate this CD8 T-cell phenotype, which in turn contributes to the development of arthritis. In this case report, the histologic features of ST were identical to those observed in RA (32), as previously shown in another case report (33). Taken together, these observations support the hypothesis of a common immune dysregulation between ICI-induced arthritis and ERA.

Beyond its clinical resemblance to RA, ICI-IA represents a rare opportunity to directly study inflamed tissue in the context of immune-related adverse events (irAEs). Compared to other irAEs—such as pneumonitis, colitis, or myocarditis—arthritis can be studied more easily due to the safety, feasibility, and reproducibility of accessing the synovial membrane. In this setting, the study of synovial tissue in ICI-IA offers a valuable model for investigating local immune dysregulation triggered by ICIs. Ultrasound-guided synovial biopsy (USGSB), a minimally invasive and widely adopted procedure in major academic centers, enables direct sampling of affected tissue, allowing for in-depth immunopathological analyses that are rarely feasible in other irAEs.

We acknowledge several limitations in our study. First, the low numerosity of the cohort necessarily impacts the statistical analysis when compared with the ERA cohort; however, in terms of clinical presentation, our cohort is in line with several previous cohorts, assuring its good representativity.

A potential limitation directly linked to patient enrollment is the possibility of referral bias, as some patients may not have been evaluated due to prior corticosteroid treatment, comorbidities (e.g., anticoagulants), or delayed referral; moreover, glucocorticoids are sometimes administered together with chemotherapy and immunotherapy, further complicating patient inclusion.

Second, we compared ICI-IA with a sex- and age-matched ERA cohort. RA is a female-gender-unbalanced disease, while ICI-IA is slightly more common in men^5^. Moreover, ERA tends to be often seropositive for RF and ACPAs. However, our primary objective was to compare ST tissue and male/female histology differences that had never been demonstrated in RA. Third, our study has some limitations, such as the absence of ultrasound features of ICI-IA compared to RA, since we did not systematically perform power Doppler evaluation in all joints. However, by selecting at least a Grade 2 synovitis according to the synovial hypertrophy score, we ensure a representative tissue collection of the synovial inflammatory process. Finally, histology and IHC have limited power in decrypting underlying pathogenic mechanisms, as already demonstrated in RA, and we cannot speculate on similarities between ICI-IA and RA. Histology has low specificity, and our study is not sufficient to propose a biological overlap between the two diseases. More mechanistic studies, including transcriptomic ones, are necessary to confirm these hypotheses.

In conclusion, for the first time, we performed a histological analysis of ST from a cohort of ICI-IA patients. When comparing it with untreated ERA, we showed a similar histological profile.

## Data Availability

The raw data supporting the conclusions of this article will be made available by the authors, upon reasonable request.

## Glossary

ICIs: immune checkpoint inhibitors
ICI-IA: immune checkpoint inhibitor-induced arthritis
irAEs: immune-related adverse event
US: ultrasound
USBSB: ultrasound-guided synovial biopsy
RA: rheumatoid arthritis
PD1: programmed death 1
PD1 L: programmed death 1 ligand
AID: autoimmune diseases
TJC: tender joint count
SJC: swollen joint count
DAS28: Disease Activity Score 28 joints
CRP: C-reactive protein
ACPA: anti-citrullinated protein antibodies
ANA: anti-nuclear antibodies
ANCA: anti-neutrophil cytoplasm antibodies
NSCLS: non-small-cell lung carcinoma
SCLS: small-cell lung carcinoma
HCC: hepatic carcinoma
RF: rheumatoid factor
MCP: metacarpophalangeal joint
PIP: proximal interphalangeal joint
MTP: metatarsophalangeal joint
CTLA4: cytotoxic T-lymphocyte-associated protein 4
ERA: early rheumatoid arthritis
DM: diffuse-myeloid
LM: lympho-myeloid
PI: pauci-immune
HAQ: Health Assessment Questionnaire
