# Supplementary material for: Immune checkpoint-induced arthritis: a comprehensive single-cohort descriptive analysis from clinical evaluation to histology
Source: Front Med (Lausanne). 2025 Oct 13;12:1638139. doi: 10.3389/fmed.2025.1638139 (PMC12554697; doi:10.3389/fmed.2025.1638139)
Supplement: Supplementary file 1 [file Table_1.DOCX]

|  | **TJC** | **SJC** | **CRP (mg/dL)** | **DAS28** |
| --- | --- | --- | --- | --- |
| **Lining hyperplasia** | r =- 0.05 [-0,69 to 0,63]; p= 0.90 | r = [-0,59 to 0,78] ; p= 0.67 | r = [-0,87 to 0,23] ; p= 0.17 | r = [-0,84 to 0,46] ; p= 0.38 |
| **Fibrinoid necrosis** | r = 0.51[-0,22 to 0,87] ; p= 0.16 | r = [-0,53 to 0,81] ; p= 0.52 | r = [-0,26 to 0,86] ; p= 0.19 | r = [-0,56 to 0,80] ; p= 0.57 |
| **Inflammatory infiltrate** | r =0.16 [-0,56 to 0,74] ; p=0.67 | r = [-0,81 to 0,53] ; p= 0.51 | r = [-0,25 to 0,87] ; p= 0.18 | r = [-0,69 to 0,71] ; p= 0.95 |
| **Hypervascularization** | r = 0.18 [-0,55 to 0,75] ; p=0.65 | r = [-0,46 to 0,84] ; p= 0.38 | r = [-0,80 to 0,44] ; p= 0.42 | r = [-0,52 to 0,82] ; p= 0.50 |
| **CD3** | r = 0.06 [-0,62 to 0,69] ; p=0.87 | r = [-0,73 to 0,67] ; p= 0.90 | r = [-0,33 to 0,84] ; p= 0.25 | r = [-0,61 to 0,77] ; p= 0.70 |
| **CD20** | r = -0.27 [-0,79 to 0,47] ; p=0.48 | r = [-0,74 to 0,65] ; p= 0.84 | r = [-0,80 to 0,45] ; p= 0.44 | r = [-0,84 to 0,46] ; p= 0.39 |
| **CD68** | r = -0.48 [-0,86 to 0,26] ; p=0.19 | r = [-0,81 to 0,55] ; p= 0.55 | r = [-0,87 to 0,25] ; p= 0.18 | r = -[0,93 to 0,078] ; p= 0.07 |
| **CD138** | r = 0.46 [-0,29 to 0,86] ; p=0.21 | r = [-0,60 to 0,78] ; p= 0.67 | r = [-0,07 to 0,91] ; p= 0.07 | r = -[0,30 to 0,89] ; p= 0.19 |
| **CD68Necrosis** | r= 0.15 [-0,57 to 0,73] ; p=0.70 | r = 0.10 [-0,64 to 0,75] ; p= 0.81 | r = 0.09 [-0,61 to 0,70] ; p= 0.66 | r = -0.19 [-0,78 to 0,59] ; p= 0.66 |

**Supplementary Table 1.**

**Legend:** Correlation analysis between clinical and serological variable and histological features. Spearman analysis. Results presented as: r Spearman value [Confidence Interval]; p value.
